# Relationship between Social Distancing and Admissions for Cerebrovascular Accidents at a Tertiary Medical Center during the COVID-19 Pandemic: A Retrospective, Community-Based Study

**DOI:** 10.3390/jpm12050664

**Published:** 2022-04-21

**Authors:** Shin-Woong Ko, Hyoung-Joon Chun, Hyeong-Joong Yi, Kyu-Sun Choi

**Affiliations:** Department of Neurosurgery, College of Medicine, Hanyang University, 17 Haengdang-dong, Seongdong-gu, Seoul 133-792, Korea; pakky100@naver.com (S.-W.K.); tdy815@hanyang.ac.kr (H.-J.C.)

**Keywords:** COVID-19, cerebrovascular accident, ischemic stroke, transient ischemic attack, intracerebral hemorrhage, subarachnoid hemorrhage, social distancing

## Abstract

We evaluated the trend of admission of patients with acute cerebrovascular accidents (CVAs) during social distancing measures implemented during the coronavirus disease 2019 (COVID-19) era. The data of patients admitted with transient ischemic attack, ischemic stroke, intracerebral hemorrhage (ICH), and subarachnoid hemorrhage (SAH) to the emergency department of the Hanyang University Seoul Hospital were retrospectively analyzed. The data were compared between the pre-COVID-19 and COVID-19 periods. Poisson regression analysis was performed to evaluate changes in admission rates as a function of the year, social distancing level, and the interaction between the year and social distancing level. The number of admissions for CVAs dropped from 674 in the pre-COVID-19 period to 582 in the COVID-19 period. The decline in the number of admissions for ICH during social distancing measures was statistically significant, while the declines in SAH and ischemic stroke admissions were not. When the social distancing level was raised, admissions for CVAs dropped by 19.8%. The correlation between social distancing and decreased admissions for CVAs is a paradoxical relationship that may be of interest to the field of public health.

## 1. Introduction

The coronavirus disease 2019 (COVID-19) pandemic, which emerged in Wuhan, China, in December 2019, has had a major impact on healthcare systems. To reduce the spread of the virus, numerous countries have enforced lockdowns. In South Korea, mask-wearing and social distancing were implemented to mitigate the rapid viral spread. The government has gradually tightened restrictions, and in turn, individual activity has decreased, and lifestyles have changed since the start of the COVID-19 pandemic. Subsequently, people have avoided public places, such as restaurants, department stores, sports stadiums, and, most importantly, hospitals, which has reduced the number of patients admitted to the emergency department [1]. Despite the emergency department being a place where people visit for urgent help [2], there has been a decline in admissions for acute coronary syndrome and orthopedic emergencies, as well as admissions to neurosurgery and neurology [3,4,5,6].

Global studies have indicated falling rates of cerebrovascular accidents (CVAs) [1] and decreased hospital admissions for CVAs during the COVID-19 pandemic [7,8,9,10]. However, no research on this association has been conducted in South Korea.

Herein, we evaluated the trend of admission of patients with acute CVAs during social distancing measures implemented during the COVID-19 era based on community-based tertiary medical centers.

## 2. Materials and Methods

We retrospectively analyzed the data of patients admitted to the Hanyang University Seoul Hospital. This regional emergency medical center primarily caters to patients from southeastern Seoul. Patients transferred from other regions of South Korea, especially from the nearby Gyeonggi-do Province, are also treated here. 

Patients admitted to the emergency department with ischemic stroke (I63, I64), intracerebral hemorrhage (ICH [I61, I62]), and subarachnoid hemorrhage (SAH [I60]) were included in this study. All diagnoses were made based on disease classification according to the International Statistical Classification of Diseases and Related Health Problems, 10th revision. 

We included transient ischemic attack (TIA) as a component of ischemic stroke and ICH as a part of hemorrhagic stroke. Patients with SAH were also included in this study. Patients with traumatic brain hemorrhage were excluded from this study to ensure the evaluation of spontaneous cerebrovascular events. Each patient’s chart was reviewed to evaluate and compare risk factors for CVA. 

This study aimed to investigate COVID-19 in uninfected patients to reduce possible sources of variance. Therefore, in 2020, we followed the treatment guidelines recommended by the Korean Neurosurgical Society during the COVID-19 pandemic, and none of our patients tested positive for the disease. 

The period from 1 January to 31 December 2019 was considered the pre-COVID-19 period, whereas 1 January to 31 December 2020 was considered the COVID-19 period. The number of patients confirmed to have COVID-19 was released by the Korean government [11].

In 2020, there were three COVID-19 outbreaks, and the government increased the social distancing level each time (Table 1) [12,13]. We categorized these levels based on the time period and designated them as strength 1 (18 February to 5 May; social distancing levels were yet to be defined; instead, strongest social distancing came into effect), strength 2 (12 August to 12 November; three levels of national social distancing were defined), and strength 3 (13 November and onward; four levels of social distancing were defined).

Considering several diseases wax and wane with the seasons, this factor was included in the analysis. The seasons’ definition was followed by the previous study as spring (March to May), summer (June to August), fall (September to November), and winter (December to February) [14]. These seasons are referred to as Korean climate change.

**Table 1:** Key points of the revised social distancing plan [15].

Classification	Level 1	Level 2	Level 3	Level 4
Definition	Contained and stable	Local transmission/cap on gathering size	Regional transmission/ban on gatherings	Full-blown nationwide transmission/ban on going out
Decision/ adjustment authorities	*Si/gun/gu* *, city/province, CDSCH	*Si/gun/gu* *, city/province, CDSCH	*Si/gun/gu* *, city/province, CDSCH	CDSCH
Criteria	<1 case/100,000 people (weekly average) Nationwide: <500 Greater Seoul: <250	≥1 case/100,000 people (weekly average > threshold for 3+ days) Nationwide: ≥500 Greater Seoul: ≥250	≥2 cases/100,000 people (weekly average > threshold for 3+ days) Nationwide: ≥1000 Greater Seoul: ≥500	≥4 cases/100,000 people (weekly average > threshold for 3+ days) Nationwide: ≥2000 Greater Seoul: ≥1000
Private gatherings	Comply with COVID-19 protocols	Up to 8 people (gatherings of 9+ prohibited)	Up to 4 people (gatherings of 5+ prohibited)	Up to 2 people after 18:00 (gatherings of 3+ prohibited) * Private gatherings of up to 4 persons permitted until 18:00
	Fully/partially vaccinated people are not counted for immediate family gatherings Fully vaccinated people are not counted for private gatherings (except level 4)			
Events	500+ people only with advance reporting to local authorities	100+ people prohibited	50+ people prohibited	Events prohibited
	Fully vaccinated people are not counted for events			
Assemblies	500+ people prohibited	100+ people prohibited	50+ people prohibited	Prohibited except for 1-person protests
	Fully vaccinated people are not counted for assemblies			

* Si/gun/gu, administrative divisions of South Korea; CDSCH, Central Disaster and Safety Countermeasures Headquarters.

### Statistical Analysis

A Poisson regression analysis was used to test whether the rate of admissions changed during the strength 1, 2, and 3 periods. As social distancing was implemented in 2020, this logistic was applied only to the 2020 data using the formula:

$$\log\left(\lambda_{i}\right)=\beta_{0}+\beta_{1}Year_{i}+\beta_{2}Season_{i}+\beta_{3}Distance_{i}$$

The change in admission rate in association with seasonal variation was analyzed using the Poisson regression model as follows:

$$\log\left(\lambda_{i}\right)=\beta_{0}+\beta_{2i}Year_{i}\times Season_{i}$$

These regression models suggest that by exponentiating the coefficient on Year and Season, we obtain the multiplicative factor by which the count changes.

The mean monthly admission numbers were compared using the paired t-test and reported as means with standard deviations. Differences were considered statistically significant at *p* < 0.05. All statistical analyses were conducted using R software, version 4.1.2 downloadable at http://cran.r-project.org/ (accessed on 1 December 2021) (R Foundation for Statistical Computing, Vienna, Austria).

## 3. Results

The first case of COVID-19 in South Korea was reported in January 2020. The pandemic has affected healthcare centers worldwide, and each country’s government is implementing measures to arrest the spread of this disease. In South Korea, social distancing was implemented to mitigate the viral spread. During rapid increases in COVID-19 cases, the government tightened its restrictions with additional rules, such as prohibiting group meetings and restricting the maximum number of people using restaurants. Clubs and gyms faced similar restrictions, and religious activities were prohibited. Furthermore, numerous companies instituted telecommuting, which led to lifestyle changes in the population.

**Figure 1:**
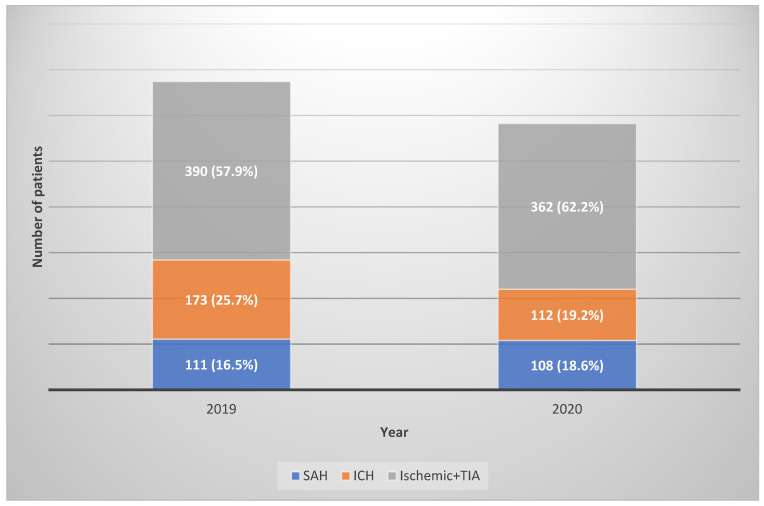
Comparison of admissions for cerebrovascular accidents between 2019 and 2020. TIA, transient ischemic attack.

From January to December 2019, 674 patients (mean age, 65.38 years; 47% women) with ischemic stroke, ICH, or SAH were admitted to the emergency room (Table 2). The mean monthly admission number was 56.17 ± 7.6. In 2020, the total number of admissions dropped to 582, and the mean monthly admission number also declined to 48.5 ± 9.68 (Table 2, Figure 1). There were no significant differences in patient characteristics between the groups. 

Of the total cases in 2019, ischemic stroke, including TIA, accounted for 57.9%; ICH, 25.7%; and SAH, 16.5%. In 2020, the corresponding values were 62.6%, 19.2%, and 18.6%, respectively (Figure 1).

The overall trend for all three CVA groups showed decreased admission rates; however, this was only statistically significant in the ICH group (Table 3).

**Figure 2:**
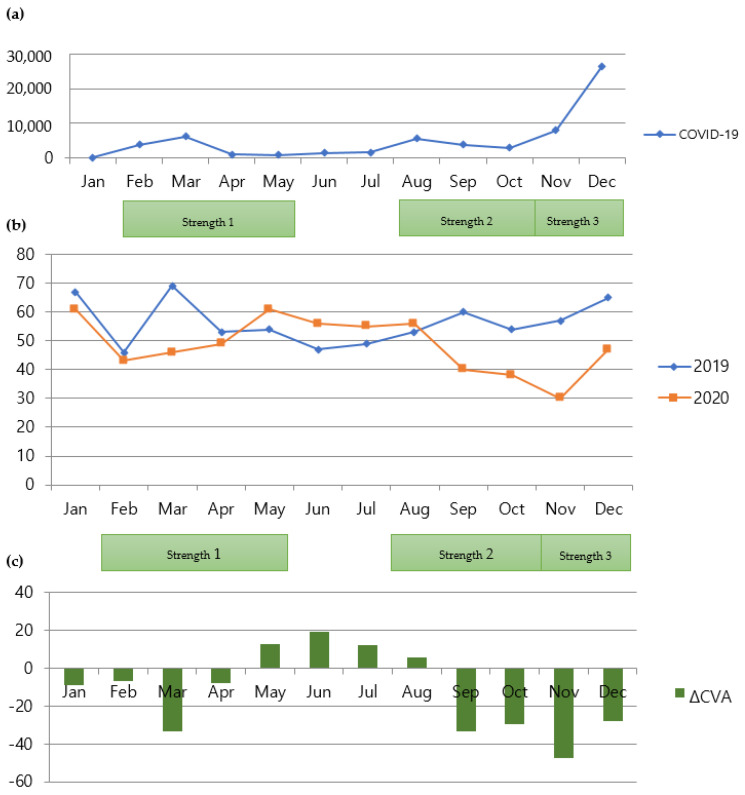
Relationship between COVID-19 and admissions for CVA. (**a**) Monthly numbers of patients with COVID-19 in Seoul, South Korea; (**b**) comparison of monthly numbers of patients admitted for CVA between 2019 and 2020 (cases of traumatic brain injury were excluded); (**c**) comparison of monthly variations in numbers of patients with CVA between 2019 and 2020. COVID-19, coronavirus disease 2019; CVA, cerebrovascular accident.

During social distancing measures, the number of admissions for CVA was significantly affected by a factor of year, which dropped by 13.65% (rate ratio is exp [−0.14676] = 0.86350) (Table 4, *p* = 0.0095). Moreover, the number of admissions was significantly affected by a factor of the season fall. It dropped by 15.97% (rate ratio is exp [−0.173923] = 0.84036) compared to spring. However, it is not significantly different from other seasons. Figure 2 shows the comparison of monthly admission numbers.

**Figure 3:**
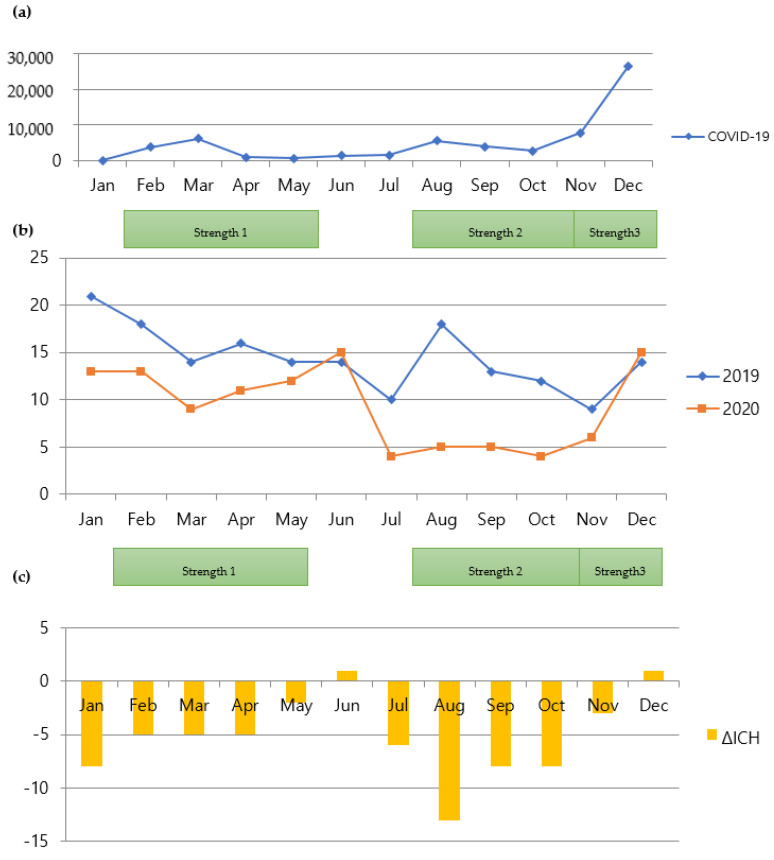
Relationship between COVID-19 and admissions for ICH. (**a**) Monthly numbers of patients with COVID-19 in Seoul, South Korea; (**b**) comparison of monthly admissions for spontaneous ICH between 2019 and 2020; (**c**) comparison of monthly variations in numbers of patients with ICH between 2019 and 2020. COVID-19, coronavirus disease 2019; ICH, intracerebral hemorrhage.

**Figure 4:**
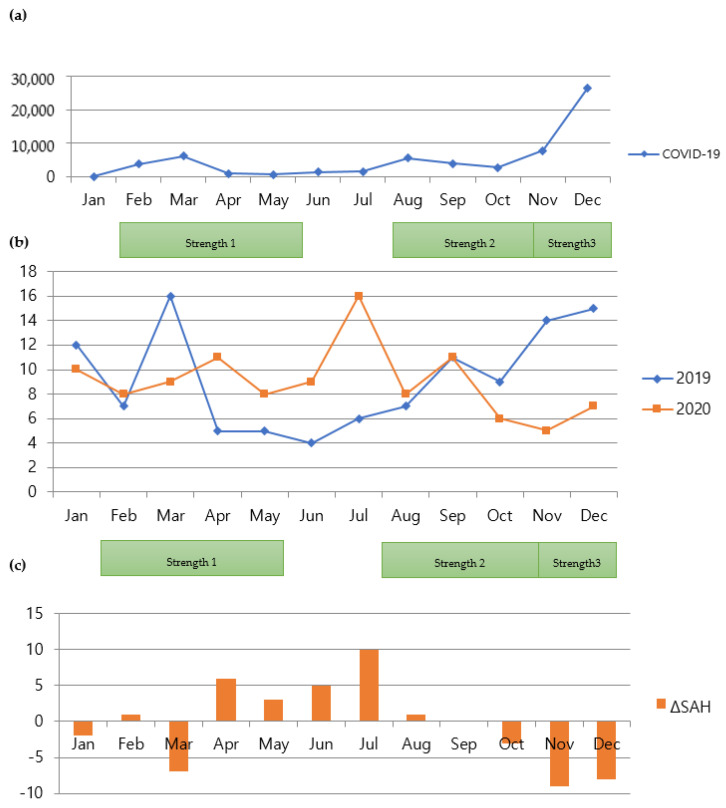
(**a**) Monthly numbers of patients with COVID-19 in Seoul, South Korea; (**b**) comparison of monthly admissions for SAH between 2019 and 2020; (**c**) comparison of monthly variations in numbers of patients with SAH between 2019 and 2020. COVID-19, coronavirus disease 2019; SAH, subarachnoid hemorrhage.

**Figure 5:**
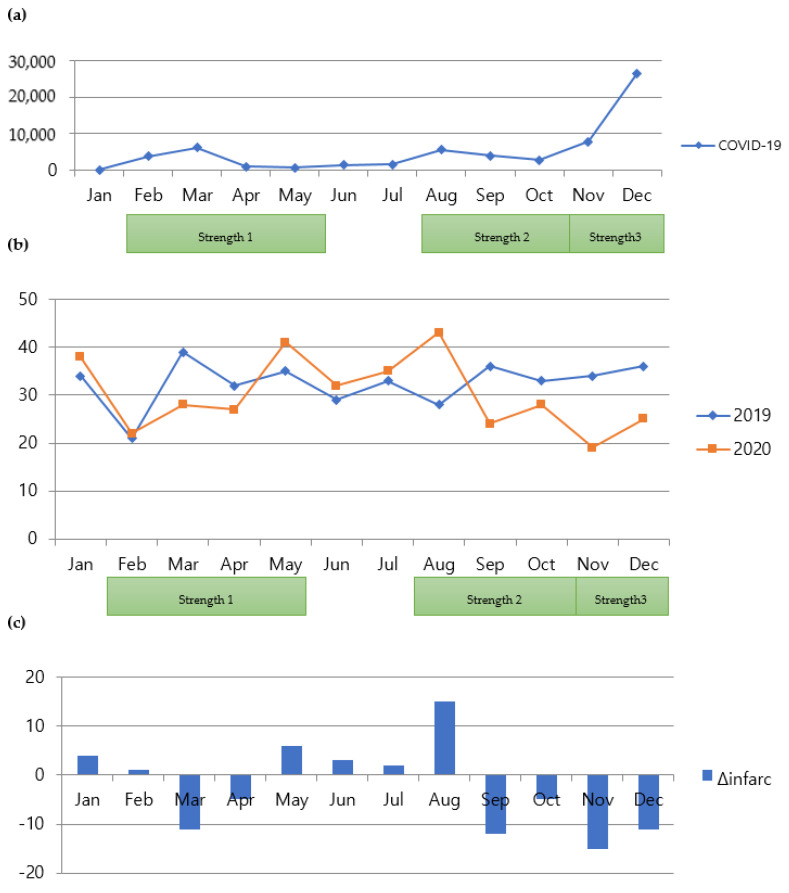
(**a**) Monthly numbers of patients with COVID-19 in Seoul, South Korea; (**b**) comparison of monthly admissions for ischemic stroke between 2019 and 2020; (**c**) comparison of monthly variations in numbers of patients with ischemic stroke between 2019 and 2020. COVID-19, coronavirus disease 2019.

While all the CVA groups demonstrated decreased admissions during social distancing, the number of admissions for ICH was significantly affected by the factor of Year, which dropped by 35.3% (rate ratio is exp (−0.4348) = 0.64739) (Table 5, *p* = 0.000337). Furthermore, the number of admissions was significantly affected by the factor of the season fall. The number dropped by 35.53% (rate ratio is exp (−0. 4389) = 0.64475) compared to spring. However, it was not significantly different from other seasons. Figure 3 shows the comparison of monthly admission numbers. However, these findings are different from those of a previous study [14]. Figure 4 and Figure 5 show the monthly variations in each group. 

In 2020, when social distancing levels were raised, the admission numbers dropped by 19.8% (*p* = 0.02435). Moreover, the admission number decreased by 27.3% in the winter (*p* = 0.00629, Table 6). When the time periods corresponding to strengths 1, 2, and 3 were studied, the strength 1 period showed the largest decline of 34.2% (*p* = 0.0014, Table 7).

## 4. Discussion

Previous studies have reported a decreased number of patients visiting emergency rooms during the COVID-19 era [16], including transient ischemic attacks and strokes [10]. We confirmed that the COVID-19 pandemic was associated with decreased mean monthly admissions for patients with acute CVA. Although there was no significant difference in patient characteristics between different types of strokes, stricter social distancing measures were associated with decreased admission rates of patients with CVAs, especially ICH. However, the reductions in ischemic stroke and SAH admissions were not statistically significant, contradicting previous findings, which showed decreased demand for care [17]. A recent meta-analysis reported that SAH occurrence was low in summer than in winter months [18]. Paradoxically, during the COVID-19 era, our data show that CVA incidents were lowest during the winter months.

In 2020, there were three waves of COVID-19 in South Korea, and the levels of social distancing were increased each time; we analyzed admissions for CVAs during these periods designated as strengths 1, 2, and 3. On implementing social distancing measures, the number of admissions decreased compared with that during the rest of the year. In the strength 1 period, there was the greatest reduction in admissions. 

Based on our results, we may think of the extra effect of social distancing. During the rapid increase in COVID-19 cases, the government tightened its restrictions, decreasing individual physical activity. Physical activity is well known for preventive measures for ischemic stroke [19]. The decrease in admissions for CVA during the COVID-19 era presents a paradox because the COVID-19 pandemic resulted in decreased levels of physical activity. Potential explanations for this discrepancy include increased happiness during the COVID-19 pandemic, reduced stress [20] and decreased social activity [21].

Our study had a limitation; namely, it was a single-center study. Nevertheless, considering that our hospital is a community-based tertiary care center and a regional emergency medical center that covers Southeastern Seoul, the changing trend of admissions that we observed could represent the overall pattern in South Korea. However, further studies need to be conducted, including more recent data and multiple medical centers, to validate our findings.

## 5. Conclusions

The onset of COVID-19 has made the South Korean government implement mask-wearing and social distancing to mitigate the viral spread. In South Korea, and likewise in other countries worldwide, acute stroke admissions have dropped in the COVID-19 era. Especially when social distancing levels were strict, the admission number of strokes dropped significantly. Several vaccines against COVID-19 have been approved in South Korea, and vaccination drives have been conducted. By steadily increasing these rates, the South Korean government is now preparing to adopt the ‘With COVID-19’ scheme as soon as 80% of adults are fully vaccinated. Based on this plan, the government seeks a gradual return to normal life with relaxed social distancing measures. This study demonstrates that stringent social distancing measures are correlated with a decrease in admissions for CVAs at a regional hospital in South Korea. Our findings highlight a paradoxical relationship that may be of interest to the field of public health.

## Figures and Tables

**Table 2:** Characteristics of patients admitted for cerebrovascular accidents in 2019 and 2020.

	2019 (*n* = 674)	2020 (*n* = 582)	*p* Value
Age, mean ± SD *, y	65.38(14.72)	65.18(15.09)	0.821
Women, *n* (%)	316 (0.47)	265 (0.46)	0.6728
Hypertension, *n* (%)	376 (0.56)	325 (0.56)	1
Hyperlipidemia, *n* (%)	207 (0.31)	194 (0.33)	0.3508
Diabetes mellitus, *n* (%)	173 (0.26)	138 (0.24)	0.4621
Heart failure, *m* (%)	23 (0.03)	8 (0.01)	0.02345
Arrythmia, *n* (%)	72 (0.11)	42 (0.07)	0.04198
Chronic renal failure, *n* (%)	30 (0.04)	28 (0.05)	0.8663
Antiplatelet agent usage, *n* (%)	145 (0.22)	130 (0.22)	0.7768
Anticoagulant usage, *n* (%)	27 (0.04)	25 (0.04)	0.9085
Smokers, *n* (%)	261 (0.39)	224 (0.38)	0.978
Mean monthly admissions, mean ± SD *, *n*	56.17 (7.65)	48.5 (9.68)	0.06018

* SD, standard deviation.

**Table 3:** Characteristics of patients admitted for ICH, ischemic stroke and SAH in 2019 and 2020.

	Disease								
	Intracerebral Hemorrhage			Ischemic Stroke			Subarachnoid Hemorrhage		
	2019 (*n* = 173)	2020 (*n* = 112)	*p* Value	2019 (*n* = 390)	2020 (*n* = 362)	*p* Value	2019 (*n* = 111)	2020 (*n* = 108)	*p* Value
Age, mean ± SD *, y	61.52 (15.97)	61.55 (14.2)	0.9853	69.04 (13.1)	68.43 (14.57)	0.5542	58.54 (14.22)	58.06 (14.49)	0.8067
Female, *n* (%)	71 (41%)	42 (38%)	0.6363	178 (46%)	147 (41%)	0.1873	67 (60%)	76 (70%)	0.1574
Hypertension, *n* (%)	85 (0.49)	66 (0.59)	0.1345	244 (0.63)	221 (0.61)	0.7248	47 (0.42)	38 (0.35)	0.3432
Hyperlipidemia, *n* (%)	32 (0.18)	28 (0.25)	0.2434	156 (0.40	145 (0.40)	1	19 (0.17)	21 (0.19)	0.7866
Diabetes mellitus, *n* (%)	34 (0.20)	17 (0.15)	0.4212	126 (0.32)	110 (0.30)	0.6252	13 (0.12)	11 (0.10)	0.8845
Heart failure, *m* (%)	4	0	-	19 (0.049)	8 (0.02)	0.0777	0	0	-
Arrythmia, *n* (%)	13 (0.08)	3 (0.03)	0.1419	57 (0.15)	39 (0.11)	0.1421	2 (0.02)	0	0.4896
Chronic renal failure, *n* (%)	9 (0.05)	6 (0.05)	1	18 (0.05)	21 (0.06)	0.57	3 (0.03)	1 (0.01)	0.6333
Antiplatelet agent usage, *n* (%)	30(0.17)	20 (0.18)	1	109 (0.28)	100 (0.28)	0.9858	6 (0.05)	10 (0.09)	0.4032
Anticoagulant usage, *n* (%)	6 (0.03)	3 (0.03)	0.9796	19 (0.05)	20 (0.06)	0.8111	2 (0.02)	2 (0.02)	1
Smokers, *n* (%)	68 (0.39)	35 (0.31)	0.2089	152 (0.39)	153 (0.42)	0.3987	41 (0.37)	36 (0.33)	0.6768
Mean monthly admissions, *n*, mean ± SD *	14.4 (3.42)	9.3 (4.33)	0.001079	32.5 (4.70)	30.2 (7.66)	0.3873	9.3 (4.22)	9.0 (2.86)	0.8853

* SD, standard deviation.

**Table 4:** Poisson regression analysis of seasonal variation and year-by-year interactions in patients with cerebrovascular accidents.

	Estimate	Exp *	Standard Error	z Value	Pr (>|z|)
Year 2020	−0.14676	0.86350	0.056585	−2.594	0.0095
Summer	−0.049393	0.9518	0.078591	−0.628	0.5297
Fall	−0.173923	0.84036	0.081217	−2.141	0.0322
Winter	−0.009077	0.99096	0.077792	−0.117	0.9071

* Exp, exponential value.

**Table 5:** Poisson regression analysis of seasonal variation and year-by-year interactions in patients with intracerebral hemorrhage.

	Estimate	Exp *	Standard Error	z Value	Pr (>|z|)
Year 2020	−0.4348	0.64739	0.1213	−3.585	0.000337
Summer	−0.1411	0.8684	0.1683	−0.838	0.401758
Fall	−0.4389	0.64475	0.1832	−2.396	0.01659
Winter	0.2126	1.23689	0.1543	1.378	0.168221

* Exp, exponential value.

**Table 6:** Poisson regression analysis of seasonal variation and year-by-strength interactions in 2020.

	Estimate	Exp ^†^	Standard Error	z Value	Pr (>|z|)
Summer	−0.04039	0.9604148	0.11899	−0.339	0.73432
Fall	−0.24955	0.7791513	0.13167	−1.895	0.05806
Winter	−0.31829	0.7273918	0.11649	−2.732	0.00629
Strength *	−0.22031	0.8022701	0.09785	−2.252	0.02435

* Combination of strengths 1, 2, and 3; **^†^** Exp, exponential value.

**Table 7:** Poisson regression analysis for each strength period in 2020.

	Estimate	Exp	Standard Error	z Value	Pr (>|z|)
Strength 1	−0.41838	0.6581121	0.13106	−3.192	0.001411
Strength 2	0.01326	1.013348	0.14742	0.09	0.928324
Strength 3	−0.08163	0.9216129	0.15735	−0.519	0.603934

Strength 1: 18 February to 5 May, strength 2: 12 August to 12 November, strength 3: 13 November onward.

## Data Availability

Not applicable.
